# Pulsed Electromagnetic Field Therapy for Mild‐to‐Moderate Knee Osteoarthritis: A Double‐Blind, Randomized, Placebo‐Controlled Clinical Trial

**DOI:** 10.1002/jcsm.70199

**Published:** 2026-01-26

**Authors:** Kenney Ki Lee Lau, Abbey Ssu Chi Chen, Christine Hoi Yan Fu, Jonathan Patrick Ng, Michael Tim Yun Ong, Patrick Shu Hang Yung, Pauline Po Yee Lui

**Affiliations:** ^1^ Department of Orthopaedics and Traumatology The Chinese University of Hong Kong Hong Kong China; ^2^ InnoHK Cener for Neuromusculoskeletal Restorative Medicine Hong Kong China; ^3^ Department of Orthopaedics and Traumatology Prince of Wales Hospital Hong Kong China

**Keywords:** degenerative knee arthritis, electromagnetic fields, knee arthroses, knee osteoarthritis, knee osteoarthrosis, pulsed electromagnetic field therapy

## Abstract

**Background:**

Current treatments for knee osteoarthritis (OA) offer limited functional and structural improvements. Compared to age‐ and gender‐matched controls, patients with knee OA show a higher prevalence of muscle weakness, which negatively affects their ability to exercise—a key factor in enhancing physical mobility and delaying disease progression. Pulsed electromagnetic field (PEMF) therapy shows promise in promoting myogenesis and chondrogenesis in pre‐clinical studies. However, its effects on muscle strength and size, cartilage deterioration and overall physical function in knee OA patients remain unclear. This randomized placebo‐controlled trial aimed to evaluate the impact of PEMF therapy on knee muscle power, lean muscle mass, femoral cartilage thickness, minimum joint space width (mJSW), lower limb physical functions and knee‐specific patient‐reported outcome (PRO) in patients with refractory mild‐to‐moderate knee OA.

**Methods:**

Sixty refractory knee OA patients, aged 50 and older, with a pain score of 4 or higher were randomized to receive either PEMF or SHAM treatment for 30 min, 3 times a week over 8 weeks. Outcome measures, including muscle strength, lean muscle mass, cartilage thickness, mJSW, mobility and PRO were assessed at baseline, immediate post‐intervention and 6‐ and 12‐month post‐intervention.

**Results:**

Significant interactions were observed between PEMF therapy and time for both knee extension peak torque (*p* < 0.001) and knee flexion peak torque (*p* = 0.007). Post hoc analysis revealed that PEMF significantly increased extension peak torque compared to the SHAM group at 6‐month post‐intervention (*p* = 0.042). The PEMF group showed a 72% increase in knee extensor strength after 6 months from baseline, whereas the SHAM group only saw a 25% increase (*p* = 0.003). However, no significant difference in knee flexion peak torque was detected between the PEMF and SHAM treatments in the post hoc analysis. Nonetheless, the knee flexor strength in the PEMF group increased by 72% at 6 months from baseline, whereas the SHAM group showed only a 24% rise (*p* = 0.022). Additionally, there were no differences between the PEMF and SHAM groups in lean muscle mass, cartilage thickness, mJSW, the 6‐m walk time and the number of repetitions of chair standing in 30 s and WOMAC (all *p* > 0.05).

**Conclusions:**

An 8‐week course of PEMF therapy significantly improved the knee extension strength in refractory patients with mild to moderate knee OA at 6‐month post‐intervention, suggesting its potential to address extensor weakness in this high‐risk population. However, it did not significantly enhance knee flexion strength, lean muscle mass, cartilage thickness, mJSW, lower limb physical functions or PRO up to 12‐month post‐intervention. Further research is needed to identify treatment regimens that may promote structural and functional outcomes in knee OA patients.

**Trial Registration:** ClinicalTrials.gov: NCT05442697

## 1 Introduction

Osteoarthritis (OA) is a degenerative joint disease characterized by the gradual breakdown of cartilage, leading to pain, stiffness, swelling and reduced mobility in the affected joints. It is the most prevalent form of arthritis, with a prevalence rate of 595 million in 2020 and is projected to increase to 642 million cases globally by 2050 [1]. Among the various joints affected, the knee is the most frequently involved site, with an age‐standardized incidence of about 350 per 100 000 individuals [1]. Current conservative management options, including pharmacological and physical therapies, provide limited symptomatic relief and functional improvement, highlighting the need for innovative therapeutic modalities.

Muscle weakness is a common manifestation of knee OA. In a cross‐sectional study of community‐dwelling German women aged 70 years and older, those with knee and hip OA exhibited significantly higher rates of sarcopenia, with a prevalence of 9.1% in individuals with OA compared to 3.5% in those without [2]. Specifically, a study by Ho et al. [3] indicated that 32.8% of patients with severe knee OA experienced sarcopenia. Muscle weakness increases joint laxity, leading to abnormal movement patterns, increased wear on the cartilage and heightened pain. Additionally, it can result in uneven load distribution, which increases stress on the affected cartilage and accelerates degeneration. Muscle weakness often leads to decreased physical activity due to pain or difficulty in movement, creating a vicious cycle that further exacerbates OA symptoms. Therefore, addressing muscle strength through targeted exercise and rehabilitation is essential for managing OA and slowing its progression.

Pulsed electromagnetic field (PEMF) therapy has emerged as a promising non‐invasive treatment for various musculoskeletal disorders. It leverages low‐frequency electromagnetic energy to enhance circulation, mitigate inflammation and boost metabolism in the targeted area. Regarding its potential effects on OA, both in vitro and in vivo animal studies have demonstrated that PEMF effectively increases chondrogenesis [4], counteracts inflammation [5, 6], enhances myoblast proliferation [7, 8], accelerates muscle regeneration [9, 10] and reduces cartilage degeneration and osteophyte formation [5, 6, 11, 12, 13]. These findings underscore the therapeutic potential of PEMF in managing OA.

Despite the encouraging results from cell culture and animal studies, the impact of PEMF therapy on treating patients with knee OA remains ambiguous. Recent systematic reviews have reported positive outcomes associated with this therapy, particularly in pain reduction and improvements in quality of life in patients with knee OA [14, 15, 16]. However, these studies typically had minimal follow‐up duration, ranging from immediate post‐treatment to a maximum of 2 months after treatment. Additionally, they included unselected patients with knee OA, providing insufficient insight into the effects across different severity groups. Moreover, all previous clinical trials have focused on symptomatic relief, with no studies investigating the disease‐modifying effects of PEMF on knee OA. There is also a lack of research on the efficacy of PEMF in improving muscle strength in these patients.

This randomized controlled trial (RCT) therefore aimed to assess the efficacy of PEMF therapy on muscle strength, mass, function, cartilage thickness, minimum joint space width (mJSW) and knee‐specific patient‐reported outcome (PRO) in refractory patients with mild‐to‐moderate knee OA, with follow‐ups lasting up to 12 months after the intervention. It is hypothesized that PEMF therapy will enhance muscle strength, increase muscle mass, improve muscle function, enhance PRO and delay articular cartilage degeneration in these patients.

## 2 Methods

### 2.1 Reporting

The results were reported in accordance with the Consolidated Standards of Reporting Trials (CONSORT) guidelines.

### 2.2 Research Ethics

This single‐centre, parallel‐group, double‐blinded, placebo‐controlled, randomized trial involved knee OA patients with repeated measures over 14 months. The study received approval from the Institutional Review Board of the Joint Chinese University of Hong Kong/New Territories East Cluster (reference number: 2022.042) and was registered in a publicly accessible database (identifier: NCT05442697). Patients were recruited and assessed following the Declaration of Helsinki and the International Conference on Harmonization Good Clinical Practice (ICH‐GCP) E6 guidelines. All eligible patients provided informed consent before participating.

### 2.3 Study Design

Sixty refractory patients with mild‐to‐moderate knee OA were randomly assigned in a 1:1 ratio to either the PEMF or SHAM groups (*n* = 30/group). The therapies lasted for 8 weeks, with clinical, imaging, functional outcomes and PROs assessed at four time points: baseline (pre‐intervention), 2 months (immediate post‐intervention), 8 months (6‐month post‐intervention) and 14 months (12‐month post‐intervention).

### 2.4 Participants

Potential subjects were screened and recruited from the specialist outpatient clinic at the Prince of Wales Hospital in Hong Kong by research staff who were blinded to the group assessments. Eligible subjects had unilateral or bilateral knee OA classified as Grade 2 or 3 according to the Kellgren–Lawrence system. For patients with bilateral OA, the more painful side received the intervention. If both limbs exhibited the same level of pain, the limb with a higher Kellgren–Lawrence grade would be chosen. Eligibility required patients to be at least 50 years old, with a pain score of 4 or higher on the visual analogue scale (VAS). Candidates must not have experienced significant symptom relief from conservative management or had acute knee injuries or muscle strains within the past 3 months. Exclusion criteria included dermatological conditions affecting the knee, severe pain in other regions, recent intra‐articular injections, inflammatory or infectious joint disorders and contraindicated medical devices (e.g., pacemakers and neurostimulators). Pregnant or breastfeeding individuals, those physically unable to participate and those incapable of providing informed consent were also excluded.

### 2.5 PEMF and SHAM Interventions

Subjects in the PEMF group received active PEMF therapy, whereas those in the SHAM group underwent a sham exposure using the same PEMF device (BIXEPS, Quantum TX, Singapore). Only a single knee was exposed to the therapy. Each session lasted 30 min, with three sessions per week for 8 weeks. This regimen was chosen based on most studies reporting positive effects of PEMF at a minimum duration of 30 min [14, 15, 16]. The PEMF therapy settings of 1 mT and 50 Hz were chosen based on previous research showing that PEMF at low amplitudes of 1–1.5 mT and frequencies below 100 Hz promotes myogenesis [17, 18] and chondrogenesis [19] in cell culture studies. Furthermore, a case series without a proper control group indicated that a weekly 10‐min PEMF treatment at 1 mT over 12 weeks led to significant improvements in mobility, skeletal muscle mass and pain perception among a convenience sample of healthy volunteers and individuals with pre‐existing mobility issues [20]. During each session in our study, subjects sat in a chair with the index knee positioned in the device, where the solenoids were placed over it. With reference to previous studies, PEMF was applied at an intensity of 1 mT and a frequency of 50 Hz. The SHAM group followed the same protocol but without the PEMF signals.

### 2.6 Randomization and Blinding

Group assignment occurred post‐screening and after obtaining informed consent. Subjects were randomly assigned to the PEMF or SHAM groups in a 1:1 ratio using a block size of 10 based on an allocation sequence generated by an independent biostatistician. The biostatistician was instructed by the principal investigator not to disclose group assignments to the patients or research staff. The PEMF device was activated using a personalized card programmed for the assigned intervention. Because PEMF therapy is non‐thermal and does not produce discernible sensations, both subjects and research staff remained blinded to group assignments. In specific circumstances, such as an adverse event, the principal investigator is permitted to know the group assignment of a particular subject to provide appropriate treatment. However, no adverse effects occurred in this study.

### 2.7 Data Collection

All assessments were conducted by experienced research staff blinded to intervention allocation. Demographic information (sex, age, weight and height), medical history, pain intensity and the duration and severity of knee OA were collected during screening and at baseline. Standing x‐ray imaging was performed at baseline (pre‐intervention), 2 months (immediate post‐intervention) and 14 months (12‐month post‐intervention). Extensor and flexor muscle strengths, lower limb muscle mass, femoral cartilage thickness, lower limb physical functions and Western Ontario and McMaster Universities Arthritis (WOMAC) were assessed at baseline, immediate post‐intervention, 6‐month post‐intervention and 12‐month post‐intervention.

### 2.8 Outcome Measures

#### 2.8.1 Muscle Strength

Isometric strengths of knee muscles were the primary outcomes. A handheld dynamometer (MicroFET2, Hoggan Scientific, USA) measures muscle strength, showing high intra‐rater reliability (ICC values > 0.89 for both extensors and flexors [21]). Subjects, seated with legs suspended, were instructed to exert maximal effort for 5 s while stabilizing themselves by grasping the table. The dynamometer was positioned either anteriorly or posteriorly to the distal tibia above the malleoli to assess maximal voluntary isometric contractions during lower limb extension and flexion. Peak forces generated were recorded.

#### 2.8.2 Dual‐Energy X‐Ray Absorptiometry (DXA)

Lean muscle mass of the index leg was assessed using the DXA scan (Horizon, Hologic, USA). Subject lay supine during the scan, with the system automatically generating data on lean muscle mass without fat and bone.

#### 2.8.3 Sonography

Femoral cartilage thickness of the index knee was evaluated using ultrasound imaging (Aixplorer Ultimate, SuperSonic Imagine, France), validated against magnetic resonance imaging (*R* ^2^ = 0.55) [22]. Inter‐rater reliability for measurements against a sonographer was 0.981, and test–retest reliability was 0.836. Subjects were positioned supine with the knee maximally flexed while scanned with an L18‐5 MHz linear transducer over the suprapatellar region. Four to six images per subject were captured, with three clear images used to measure the maximal thickness of the medial condyle of the femur, intercondylar area of the femur and lateral condyle of the femur [23]. Cartilage thickness was measured from the first bright pixel at the lowest or highest point to the first bright pixel on the opposite side, and the average of three images was recorded.

#### 2.8.4 X‐Ray Radiography

Standing x‐rays assessed eligibility based on the Kellgren–Lawrence system and mJSW. mJSW was calculated as the distance between the tibia and femur in the medial knee compartment at 0.25 times the tibial width [24].

#### 2.8.5 Functional Tests

The 6‐m timed walking test and 30‐s chair stand test evaluated lower limb function with high reproducibility (ICC > 0.98 and > 0.90 [25, 26], respectively). For the 6‐m timed walking test, subjects walked on a 6‐m walkway at their normal pace, with time recorded by a stopwatch. Gait speed was calculated. In the chair stand test, subjects sat with arms crossed and feet shoulder–width apart and performed as many full stands as possible in 30 s. The total number of repetitions of chair standing was documented.

#### 2.8.6 WOMAC

The WOMAC questionnaire contains 24 questions with three subscales: pain, stiffness and physical function. The total score ranges from 0 to 96, with a higher score indicating severe symptoms.

#### 2.8.7 Demographic and Clinical Data

Demographic data (gender, age, height, weight and body mass index) were obtained from electronic medical records. Pain intensity (VAS 0–10) and symptom duration were collected during baseline assessment.

### 2.9 Sample Size

Sample size was calculated based on knee extensor peak torque using the G*Power Version 3.1. An interim analysis of the first 10 subjects post‐intervention informed effect sizes. For 2 comparison groups, 4 repeated measurements, a correlation of 0.349, alpha (type I error) at 0.05, power at 0.8, and effect sizes at 0.536, respectively, a total of 9 subjects per group were required to detect significant differences in extensor peak torques. To account for the potential dropouts, a total of 22 subjects were required.

### 2.10 Data Handling and Statistical Analysis

Data were analysed following the intention‐to‐treat principle, with missing data filled in using the last‐observation carry‐forward principle. Results are presented as mean ± standard deviation (SD) for data in ratio and interval scales and frequency (percentage) for data in nominal scale. All measurements, except mJSW, were taken in triplicate, and averages were used for analysis. Baseline demographic data and outcomes between the PEMF and SHAM groups were compared using an independent samples *t*‐test or a chi‐squared test. The effects of intervention and time on extensor and flexor muscle peak torques, lean muscle mass, cartilage thickness, mJSW, physical functions, KOOS and WOMAC were evaluated using two‐way repeated measures ANOVA, with post hoc Bonferroni correction to assess both main and interactive effects. A sensitivity analysis was also conducted on primary outcomes by excluding all participants with missing data. Data analysis was conducted with SPSS Version 29.0, and significance was set at *p* < 0.050 (two‐tailed).

## 3 Results

### 3.1 Participant Flow

**FIGURE 1:**
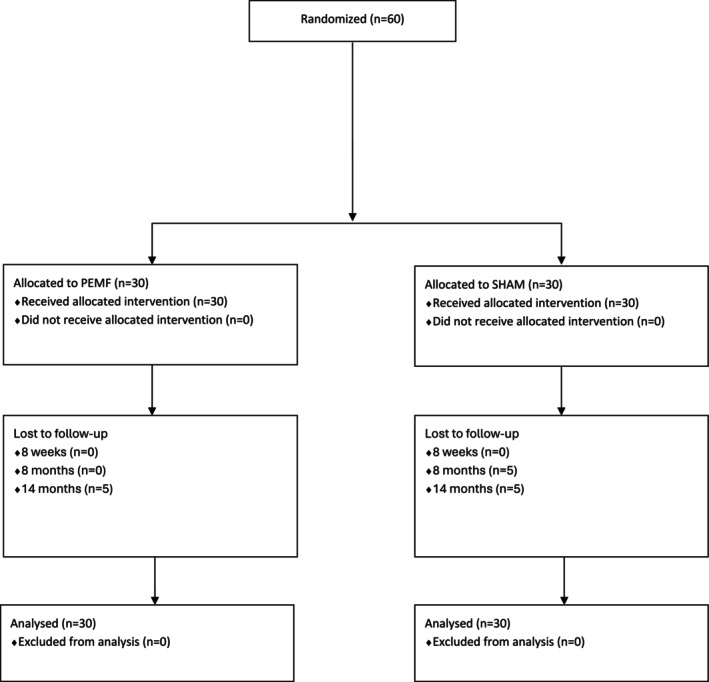
CONSORT flow diagram of number of subjects allocated to each group, follow‐up and analysed.

All individuals adhered to their assigned interventions (Figure 1). There was no decline in follow‐up immediately post‐intervention. However, there were missing data in the SHAM group at 6‐month post‐intervention (*n* = 5) and in the PEMF group at 12‐month post‐intervention (*n* = 5). The primary reasons for discontinuation included transportation challenges and loss to follow‐up. All data were included in the final analysis.

### 3.2 Baseline Data

In our knee OA cohort, 65% were female, with a mean age of 67.5 ± 5.8 years. Their average height, weight and body mass index were 1.6 ± 0.1 m, 66.9 ± 13.0 kg and 26.2 ± 3.9 kg/m^2^, respectively. A substantial majority (85%) were classified as Grade 2 according to the Kellgren and Lawrence grading system for osteoarthritis. The average pain intensity was 5.6 ± 1.5 points, and the mean duration of symptoms was 6.8 ± 4.5 years. Notably, there were no differences in demographic, clinical characteristics, imaging results, functional outcomes and PRO at baseline between the PEMF and SHAM groups (Table 1).

**TABLE 1:** Demographics and baseline characteristics of participants. Between‐group comparisons were performed using independent samples *t*‐test (continuous data) or chi‐squared test (categorical data).

	PEMF (*n* = 30)	SHAM (*n* = 30)	*p*
Demographics			
Sex	73% females	57% females	0.279
Age (years)	66.3 ± 5.2	68.7 ± 6.3	0.113
Height (m)	1.6 ± 0.1	1.6 ± 0.1	0.579
Weight (kg)	66.6 ± 14.1	67.2 ± 12.1	0.856
Body mass index (kg/m^2^)	26.2 ± 3.7	26.2 ± 4.1	0.978
Disease severity	87% KL grade 2	83% KL grade 2	0.999
Pain intensity	6.0 ± 1.6	5.3 ± 1.3	0.079
Symptom duration (years)	7.1 ± 4.7	6.5 ± 4.4	0.610
Muscle strength			
Knee extension peak torque of treated leg (kg)	12.56 ± 4.92	15.07 ± 7.11	0.118
Knee flexion peak torque of treated leg (kg)	8.98 ± 3.32	10.87 ± 4.86	0.085
Dual‐energy x‐ray absorptiometry			
Lean muscle mass of treated leg (g)	5520.96 ± 1544.82	5755.06 ± 1323.02	0.531
Sonography			
Medial condyle of femur in treated leg (cm)	0.14 ± 0.04	0.14 ± 0.05	0.582
Intercondylar area of femoral cartilage in treated leg (cm)	0.21 ± 0.06	0.21 ± 0.05	0.982
Lateral condyle of femur in treated leg (cm)	0.14 ± 0.05	0.14 ± 0.05	0.979
X‐ray radiography			
Medial joint space width of treated leg (mm)	4.69 ± 1.63	4.56 ± 2.55	0.810
Lateral joint space width of treated leg (mm)	5.68 ± 1.06	5.64 ± 1.17	0.891
Patient‐reported outcome			
WOMAC total score	40.21 ± 17.71	34.60 ± 14.58	0.217
Functional tests			
6‐m timed walking test (m/s)	1.20 ± 0.52	1.23 ± 0.37	0.799
30‐s chair stand test (count)	11.33 ± 3.91	10.03 ± 3.85	0.200

*Note:* Mean ± standard deviation or proportion in percentage.

Abbreviations: KL grade = Kellgren and Lawrence classification of osteoarthritis, PEMF = pulsed electromagnetic field therapy, SHAM = sham treatment, WOMAC = Western Ontario and McMaster Universities Arthritis Index.

### 3.3 Outcomes

#### 3.3.1 Primary Outcomes

**FIGURE 2:**
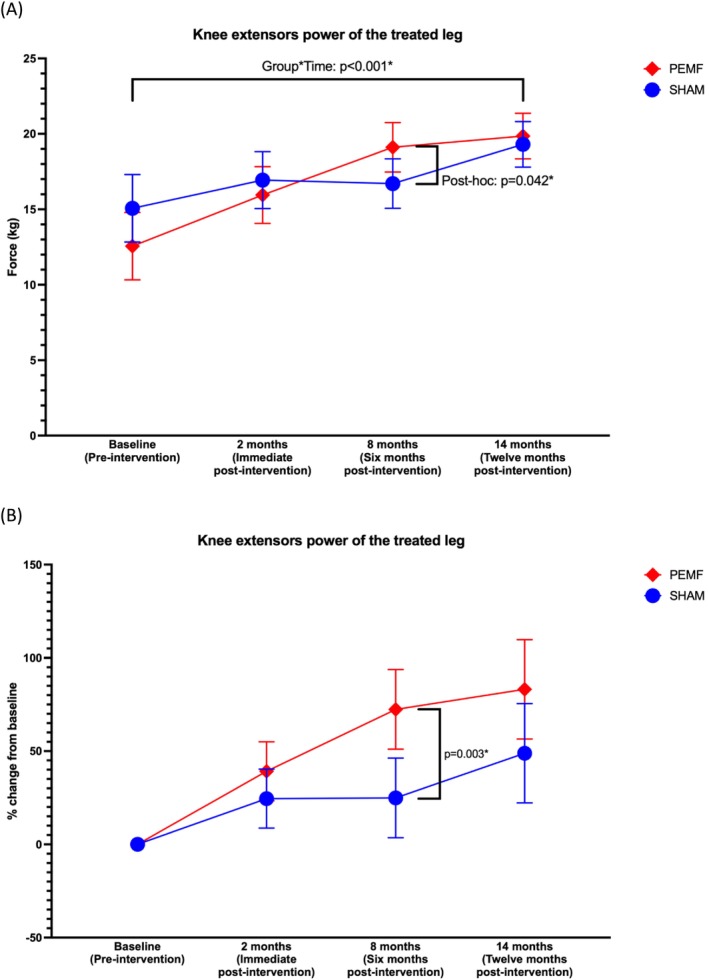
(A) Changes in knee extension peak torque of the treated limb with time in the PEMF and SHAM groups (*n* = 30/group). Analysed using a two‐way repeated measures ANOVA with two treatment groups and four time points, an interaction effect was identified. Post hoc test revealed a significant difference between groups at 6 months post‐intervention. (B) Mean percentage change from baseline of each patient at different follow‐up time points. Analysed using an independent samples *t*‐test, a significant difference between groups was observed at the 6‐month post‐intervention time point. Mean ± 95% CIs; *p < 0.05.

There were significant interactive effects for extension peak torque (*p* < 0.001; partial η^2^ = 0.115) (Tables 2, 3, 4, 5 and Figure 2A). Post hoc analysis revealed that PEMF significantly increased extension peak torque compared to the SHAM group 6‐month post‐intervention (mean difference = 2.403, 95% CI: 0.086–4.721, *p* = 0.042) (Table 4 and Figure 2A). The percentage change of knee extensor strength at 6 months compared to baseline in the PEMF group increased by 72%, whereas the SHAM group experienced only a 25% increase (*p* = 0.003) (Figures 2B and S1).

**TABLE 2:** Interactive, main and time effects of various outcomes of knee OA patients undergoing PEMF or SHAM therapies. Outcomes were analysed using a two‐way repeated measures ANOVA with two treatment groups and four time points.

	Interaction effect		Main effect		Time effect	
	*p*	partial η^2^	*p*	partial η^2^	*p*	partial η^2^
Muscle strength						
Knee extension peak torque of treated leg (kg)	< 0.001*	0.115	0.905	0.000	< 0.001*	0.413
Knee flexion peak torque of treated leg (kg)	0.007*	0.067	0.382	0.013	< 0.001*	0.351
Dual‐energy x‐ray absorptiometry						
Lean muscle mass of treated leg (g)	0.438	0.014	0.544	0.006	0.708	0.006
Sonography						
Medial condyle of femur in treated leg (cm)	0.644	0.006	0.227	0.025	0.009*	0.093
Intercondylar area of femoral cartilage in treated leg (cm)	0.719	0.005	0.705	0.002	0.079^a^	0.045
Lateral condyle of femur in treated leg (cm)	0.948	0.000	0.962	0.000	0.004*	0.116
X‐ray radiography						
Medial joint space width of treated leg (mm)	0.760	0.003	0.798	0.001	0.248	0.024
Lateral joint space width of treated leg (mm)	0.761	0.002	0.996	0.000	0.019*	0.087
Patient‐reported outcome						
WOMAC	0.268	0.025	0.098	0.053	< 0.001*	0.122
Functional tests						
6‐m timed walking test (m/s)	0.571	0.010	0.712	0.002	< 0.001*	0.186
30‐s chair stand test (count)	0.341	0.019	0.090^a^	0.049	< 0.001*	0.559

Abbreviations: PEMF = pulsed electromagnetic field therapy, SHAM = sham treatment, WOMAC = Western Ontario and McMaster Universities Arthritis Index.

^a^ Marginally insignificant.

* *p* < 0.05.

**TABLE 3:** Clinical, functional, imaging and patient‐reported outcomes of knee OA patients undergoing PEMF or SHAM therapies immediately post‐intervention. Outcomes were analysed using the post hoc test from the two‐way repeated measures ANOVA.

	PEMF (*n* = 30)	SHAM (*n* = 30)	MD (PEMF‐SHAM)	95% CI of MD	Post hoc *p*	Partial η^2^
Muscle strength						
Knee extension peak torque of treated leg (kg)	15.95 ± 5.01	16.94 ± 5.28	−0.987	−3.65–1.67	0.461	0.009
Knee flexion peak torque of treated leg (kg)	10.84 ± 3.93	11.99 ± 4.16	−1.157	−3.25–0.94	0.273	0.021
Dual‐energy x‐ray absorptiometry						
Lean muscle mass of treated leg (g)	5670.52 ± 1516.09	5727.74 ± 1608.41	−57.22	−865.01–750.57	0.888	0.000
Sonography						
Medial condyle of femur in treated leg (cm)	0.13 ± 0.05	0.14 ± 0.05	−0.01	−0.04–0.02	0.425	0.011
Intercondylar area of femoral cartilage in treated leg (cm)	0.21 ± 0.05	0.20 ± 0.07	0.01	−0.02–0.05	0.467	0.009
Lateral condyle of femur in treated leg (cm)	0.12 ± 0.05	0.12 ± 0.06	−0.01	−0.03–0.02	0.887	0.000
X‐ray radiography						
Medial joint space width of treated leg (mm)	4.65 ± 1.69	4.44 ± 2.76	0.20	−0.98–1.39	0.732	0.002
Lateral joint space width of treated leg (mm)	5.53 ± 1.22	5.54 ± 1.33	−0.02	−0.68–0.64	0.961	0.000
Patient‐reported outcomes						
WOMAC total score	33.75 ± 18.54	31.16 ± 13.34	2.59	−6.41–11.59	0.658	0.004
Functional tests						
6‐m timed walking test (m/s)	1.05 ± 0.25	1.12 ± 0.28	−0.07	−0.21–0.07	0.323	0.017
30‐s chair stand test (count)	12.43 ± 3.57	11.57 ± 3.50	0.87	−0.96–2.69	0.346	0.015

*Note:* Mean ± standard deviation.

Abbreviations: CI = confidence interval, MD = mean difference, PEMF = pulsed electromagnetic field therapy, SHAM = sham treatment.

**TABLE 4:** Clinical, functional, imaging and patient‐reported outcomes of knee OA patients undergoing PEMF or SHAM therapies at 6‐month post‐intervention. Outcomes were analysed using the post hoc test from the two‐way repeated measures ANOVA.

	PEMF (*n* = 30)	SHAM (*n* = 30)	MD (PEMF‐SHAM)	95% CI of MD	Post hoc *p*	Partial η^2^
Muscle strength						
Knee extension peak torque of treated leg (kg)	19.11 ± 4.13	16.71 ± 4.81	2.403	0.086–4.721	0.042*	0.069
Knee flexion peak torque of treated leg (kg)	13.13 ± 2.92	12.33 ± 3.55	0.795	−0.885–2.475	0.347	0.015
Dual‐energy x‐ray absorptiometry						
Lean muscle mass of treated leg (g)	5527.70 ± 1517.31	5909.22 ± 1464.76	−381.52	−1152.27–389.23	0.326	0.017
Sonography						
Medial condyle of femur in treated leg (cm)	0.12 ± 0.04	0.14 ± 0.05	−0.02	−0.039–0.007	0.169	0.032
Intercondylar area of femoral cartilage in treated leg (cm)	0.21 ± 0.06	0.20 ± 0.07	0.01	−0.029–0.038	0.782	0.001
Lateral condyle of femur in treated leg (cm)	0.13 ± 0.04	0.13 ± 0.06	0.01	−0.03–0.03	0.960	0.000
Patient‐reported outcomes						
WOMAC total score	34.46 ± 16.81	25.56 ± 12.21	8.90	0.715–17.09	0.087	0.057
Functional tests						
6‐m timed walking test (m/s)	1.06 ± 0.30	1.05 ± 0.26	0.02	−0.13–0.16	0.826	0.001
30‐s chair stand test (count)	15.70 ± 4.20	13.63 ± 3.34	2.07	0.11–4.03	0.039*	0.071

*Note:* Mean ± standard deviation.

Abbreviations: CI = confidence interval, MD = mean difference, PEMF = pulsed electromagnetic field therapy, SHAM = sham treatment.

* *p* < 0.05.

**TABLE 5:** Clinical, functional, imaging, and patient‐reported outcomes of knee OA patients undergoing PEMF or SHAM therapies at 12‐month post‐intervention. Outcomes were analysed using the post hoc test from the two‐way repeated measures ANOVA. Note. Mean ± standard deviation; PEMF = pulsed electromagnetic field therapy; SHAM = sham treatment; # marginally insignificant.

	PEMF (*n* = 30)	SHAM (*n* = 30)	MD (PEMF‐SHAM)	95% CI of MD	Post hoc *p*	Partial η^2^
Muscle strength						
Knee extension peak torque of treated leg (kg)	19.86 ± 3.65	19.30 ± 4.56	0.553	−1.581–2.687	0.606	0.005
Knee flexion peak torque of treated leg (kg)	13.19 ± 2.35	13.74 ± 3.00	−0.548	−1.941–0.845	0.434	0.011
Dual‐energy x‐ray absorptiometry						
Lean muscle mass of treated leg (g)	5534.08 ± 1405.55	5725.29 ± 1222.41	−191.21	−871.98–489.56	0.576	0.005
Sonography						
Medial condyle of femur in treated leg (cm)	0.11 ± 0.04	0.13 ± 0.05	−0.015	−0.038–0.008	0.205	0.028
Intercondylar area of femoral cartilage in treated leg (cm)	0.20 ± 0.07	0.19 ± 0.06	0.005	−0.029–0.038	0.783	0.001
Lateral condyle of femur in treated leg (cm)	0.12 ± 0.05	0.12 ± 0.06	−0.001	−0.028–0.026	0.961	0.000
X‐ray radiography						
Medial joint space width of treated leg (mm)	4.53 ± 1.71	4.43 ± 2.71	0.100	−1.071–1.271	0.865	0.001
Lateral joint space width of treated leg (mm)	5.39 ± 1.12	5.42 ± 1.40	−0.029	−0.70–0.642	0.932	0.000
Patient‐reported outcomes						
WOMAC total score	35.11 ± 17.49	27.12 ± 10.80	7.99	−0.15–16.12	0.144	0.042
Functional assessments						
6‐m timed walking test (m/s)	1.01 ± 0.26	1.03 ± 0.25	−0.03	−0.16–0.10	0.688	0.003
30‐s chair stand test (count)	16.57 ± 4.65	14.63 ± 3.96	1.93	−0.30–4.17	0.088^a^	0.049

*Note:* Mean ± standard deviation.

Abbreviations: PEMF = pulsed electromagnetic field therapy, SHAM = sham treatment.

^a^ Marginally insignificant.

**FIGURE 3:**
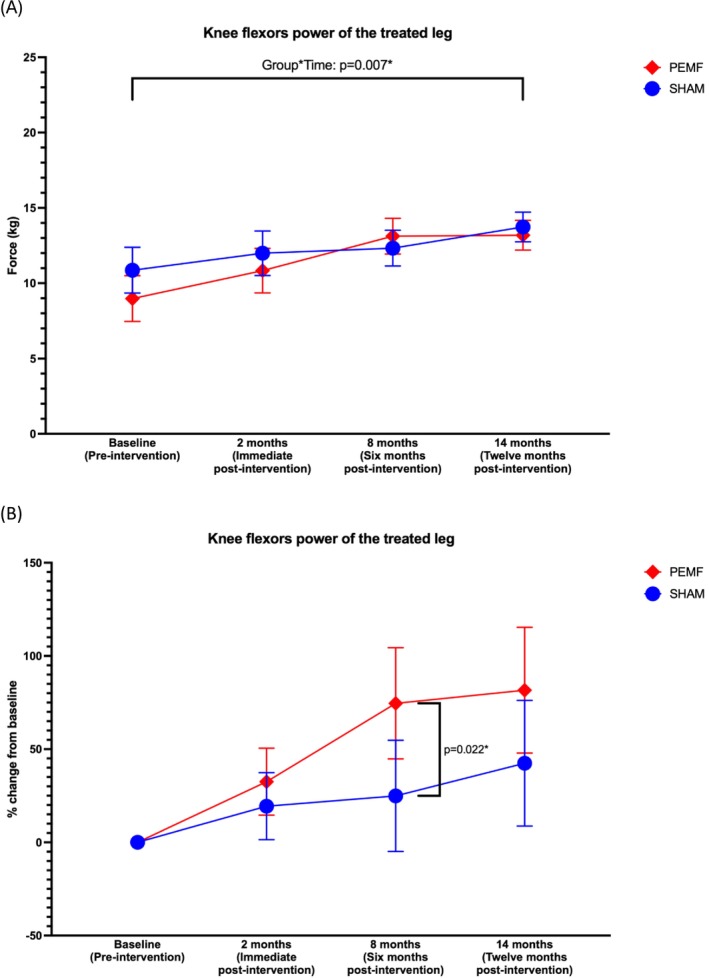
(A) Changes in knee flexion peak torque of the treated limb with time in the PEMF and SHAM groups (*n* = 30/group). Analysed using a two‐way repeated measures ANOVA with two treatment groups and four time points, an interaction effect was identified. (B) Mean percentage change from baseline of each patient at different follow‐up time points. Analysed using an independent samples *t*‐test, a significant difference between groups was observed at the 6‐month post‐intervention time point. Mean ± 95% CIs; *p < 0.05.

There were significant interactive effects for knee flexion peak torque (*p* = 0.007; partial η^2^ = 0.067) (Table 2, 3, 4, 5 and Figure 3A). However, no significant difference in flexion peak torque was detected between the PEMF and SHAM groups in the post hoc analysis (Table 3, 4, 5 and Figure 3A). Despite this, the percentage change of knee flexor strength at 6 months compared to baseline in the PEMF group rose by 72%, whereas the SHAM group exhibited only a 24% increase (*p* = 0.022) (Figure 3B and Figure S2).

#### 3.3.2 Sensitivity Analysis

Five subjects in the PEMF group and five subjects in the SHAM group dropped out at the 12‐month and 6‐month follow‐ups, respectively. There were no significant differences in patient demographics and baseline characteristics between these 10 subjects and those who remained in the study (all *p* > 0.05). An equal number of subjects dropped out from both the PEMF and SHAM groups, and there were no significant differences in outcomes between the dropouts and those who stayed in their respective groups (all *p* > 0.05), indicating that the dropouts were random. A sensitivity analysis with a reduced sample size of 50 subjects demonstrated similar significant group‐by‐time interaction effects for both knee extension peak torque (*p* = 0.012; partial η^2^ = 0.085) and knee flexion peak torque (*p* = 0.048; partial η^2^ = 0.057). However, post hoc analysis revealed no significant differences due to insufficient power.

#### 3.3.3 Secondary Outcomes

Additionally, there were no differences between the PEMF and SHAM groups in lean muscle mass, cartilage thickness, mJSW, the 6‐m walk time and the number of repetitions of chair standing in 30 s, total score of WOMAC (all *p* > 0.05) (Table 2 and Figures S3–S8). There was no significant interactive (*p* = 0.341) or group (*p* = 0.090) effect on the chair stand test (Table 2). However, there was a significant improvement in the chair stand test in the PEMF group compared to the SHAM group at 6‐month post‐intervention (mean difference = 2.07 [95% CI: 0.11–4.03]; *p* = 0.039) (Table 4).

## 4 Discussion

### 4.1 Summary

Our results indicate that PEMF therapy significantly increases extension peak torque but has no significant effects on flexor peak torque, lower limb muscle mass, cartilage thickness, mJSW, lower limb physical functions and PRO compared to SHAM therapy in refractory patients with mild‐to‐moderate knee OA.

### 4.2 Knee Extensor Strength and Clinical Implications

Our results indicate that PEMF therapy significantly enhanced knee extensor strength 6‐month post‐intervention. Specifically, the knee extensor strength in the PEMF group increased by 72% at 6 months compared to baseline, whereas the SHAM group showed only a 25% increase, indicating a significant 1.88‐fold improvement. These findings align with previous studies demonstrating the efficacy of PEMF in promoting muscle regeneration in cells [7, 8], animals [9, 10] and humans [20, 27]. For example, PEMF therapy effectively stimulated proliferation, migration and the expression of antioxidant enzymes in skeletal muscle cells [7]. It also increased myotube diameter, fusion and myosin heavy chain 1 expression of human myoblasts during myogenic differentiation [8]. In a diabetic muscle atrophy model, PEMF exposure for 30 min per day over 6 weeks significantly increased the cross‐sectional area of muscle fibres, muscle mass and muscle strength [10]. Additionally, PEMF application prevented muscle loss and maintained muscle contraction force in a disuse atrophy rat model [9]. In a clinical trial, applying PEMF (5 μT, 2 Hz) to the vastus medialis and biceps femoris during cycling augmented muscle fibre activity and glycolytic metabolism [27]. Another study reported that a weekly 10‐min PEMF treatment (1 mT, frequency unspecified) for 12 weeks significantly improved mobility, skeletal muscle mass and pain perception in a convenience sample of healthy volunteers and individuals with pre‐existing mobility dysfunction, although this study lacked a control group, which is a limitation [20]. Notably, previous preclinical and clinical studies did not specifically target muscle strength and mass in knee OA, making our study unique.

Muscle weakness can lead to joint instability and altered biomechanics, potentially increasing pain levels. This increased pain may further limit activity, exacerbating muscle weakness and contributing to OA progression. Moreover, patients with knee OA exhibit significantly higher rates of sarcopenia [2, 3]. Sarcopenic patients experience a lower quality of life [28] and higher rates of falls, fractures [29] and mortality [30]. Although regular exercise is effective for maintaining muscle mass and strength, adherence can be challenging, especially for frail and older OA patients. Therefore, PEMF therapy presents a promising non‐pharmacological and minimally invasive option to address extensor weakness in this high‐risk population. Although we observed an improvement in knee extensor strength at 6 months post‐PEMF therapy, the effect was not sustained at 12‐month post‐intervention. Longer treatment durations, as well as varying PEMF frequencies and intensities, may be necessary to maintain these benefits.

### 4.3 Knee Flexor Strength

We observed significant interactive effects of group and time on knee flexor strength. In the PEMF group, the knee flexor strength increased by 72% at 6 months compared to baseline, whereas the SHAM group showed only a 24% increase, indicating a significant twofold improvement. However, the post hoc analysis did not reveal any statistically significant improvement in the absolute value of knee flexor strength, despite the observed interactive effects. The trends in time‐dependent changes in muscle strength were similar for both knee extensors and flexors, suggesting that the insignificant differences in knee flexor strength might be attributed to substantial within‐group variation. A larger sample size may be required to confirm the effects of PEMF therapy on flexor strength.

### 4.4 Sustainability of PEMF's Effects on Muscle Strength

In our study, the percentage change in knee extensor and flexor strength increased over time following treatment. Although we observed an improvement in knee extensor strength at 6 months post‐PEMF therapy, the effect was not sustained at 12‐month post‐intervention. Gomez et al. [31] demonstrated that a 12‐month cessation of exercise during the COVID‐19 pandemic led to a significant decrease in hand muscle strength among elderly individuals who had been physically active for 10 years, with the decline observed exclusively in women. Similarly, Leitão et al. [32] found that the effects of a 9‐month exercise programme on upper and lower body strength decreased at 3 months after programme cessation and showed no significant effects compared to the non‐exercise group. However, the decrease stabilized, and muscle strength remained similar at 12 months compared to the 3‐month mark. Thus, there may be some detraining effects similar to those experienced with the cessation of physical exercise, but longer follow‐up is needed to establish a clearer pattern. Additionally, in older adults with knee OA, who often reduce their activity levels due to pain and functional limitations, the rate of decline tends to be more rapid and pronounced than in healthier populations [33]. Consequently, the attenuation of the PEMF effect at 12 months aligns with these expectations. If detraining is confirmed, longer treatment durations, as well as varied PEMF frequencies and intensities, may be necessary to maintain these benefits.

### 4.5 Muscle Mass

We did not observe any significant changes in lower limb muscle mass. In a non‐randomized study involving a small number of convenience subjects undergoing anterior cruciate ligament reconstruction (ACLR), the combination of neuromuscular electrical stimulation (NMES) and PEMF therapies demonstrated effects similar to NMES alone in reducing thigh girth loss compared to the no‐intervention group. However, the combined treatment was more tolerable and resulted in less pain 6 weeks post‐surgery. Thus, PEMF (1500 mT, 2500 Hz) did not provide additional benefits in muscle preservation in that study, which aligns with our findings [34]. It is important to note that the sample size in that study was small (*n* = 7 for the NMES/PEMF and NMES groups and *n* = 3 for the no‐intervention group), and the subjects were young patients post‐ACLR. Additionally, 4 months of PEMF therapy (1 mT, frequency unspecified) failed to improve muscle force, muscle and fat volumes compared to placebo therapy in ACLR patients, despite improvements in systemic metabolic markers [35]. Different treatment regimens, such as higher intensities, longer durations and increased frequencies, may be necessary to elicit morphological changes.

### 4.6 Physical Functions

Similarly, our findings align with prior research indicating that PEMF therapy does not enhance functional abilities [36, 37]. Although PEMF significantly improved the chair stand test at 6‐month post‐intervention compared to the SHAM intervention, there was no significant group and intervention effects. Although improvements in muscle strength were noted, these gains may not immediately translate into improved functional performance, as the body requires time to adapt and effectively incorporate increased strength into complex motor tasks [38]. Moreover, confounding variables may limit or influence the relationship between muscle strength and our selected functional assessments. For instance, timed walking performance is influenced by a combination of muscle power, cardiorespiratory fitness, motor control and dynamic balance [39]. Simply improving muscle strength may not lead to improvements in functional performance [40]. The chair stand test may also be influenced by similar factors. Consequently, it is reasonable to conclude that PEMF did not demonstrate observable improvements in overall physical functions.

### 4.7 Molecular Mechanisms and Safety of PEMF Therapy

Regarding the molecular mechanisms of PEMF, it activates calcium channel TRPC1, increases fatty acid oxidation and subsequently activates PGC‐1α, thereby promoting mitochondrial biogenesis. This process enhances the production of muscle secretome, which supports muscle maintenance and metabolism [S1].

In addition to enhancing mitochondrial biogenesis, PPARγ and its coactivator PGC‐1α exhibit significant anti‐inflammatory effects. Previous research has shown that activators of PPARγ and PGC‐1α can protect against diabetic nephropathy and suppress inflammation by inhibiting the NF‐κB signalling pathway, both in diabetic mice and in a human proximal tubular cell line [S2]. Furthermore, numerous studies have demonstrated the anti‐inflammatory effects of PEMF across various tissues, including those affected by ischemic and hypoxic conditions, cartilage and joint inflammation, as well as in venous ulcer and diabetic wound healing. These studies indicate that PEMF treatment results in the suppression of inflammatory cytokines and matrix metalloproteinases (MMPs) [S3]. Clinically, PEMF has been shown to reduce inflammation and promote healing in venous ulcers [S4, S5].

Regarding oxidative stress, a previous study has indicated that PEMF treatment at 1 mT for 4 h a day across 2 consecutive days increased the expression of various antioxidants, including thioredoxin, paraoxonases 2 and 3 (PON2, PON3), Sirtuin 2 (SIRT2), superoxide dismutase (SOD2), thioredoxin‐1, hypoxia‐inducible transcription factor 2α (HIF‐2α) and cytochrome c. This suggests that PEMF not only enhances mitochondrial function but also offers protective effects against oxidative stress and inflammation [7].

Because we did not observe any adverse effects, such as knee swelling, in our study, which is consistent with findings from other research. Based on the existing preclinical and clinical evidence, we believe that PEMF therapy is safe within the regimen we have utilized, and the likelihood of overdose or exacerbation of inflammation is minimal.

### 4.8 Clinical Significance

Knee muscle weakness is associated with pain, instability, functional decline, increased fall risk and reduced quality of life in elderly patients, particularly those with knee OA, who are especially susceptible to muscle weakness. The improvement in knee muscle strength at 6 months post‐PEMF therapy—showing a 72% increase in knee extensor strength in the PEMF group compared to only 25% in the SHAM group and a 72% increase in knee flexor strength in the PEMF group compared to just 24% in the SHAM group—despite the lack of statistically significant differences, is considered clinically important. However, the absence of sustained improvement at 12 months suggests that an 8‐week course of PEMF therapy may be insufficient for maintaining long‐term strength gains. Further studies with longer treatment durations may be necessary to sustain these benefits.

### 4.9 Effects on OA

The study of the disease‐modifying effects of PEMF therapy in a clinical setting makes our research original. We found that PEMF therapy did not significantly improve joint space narrowing, cartilage thickness or PRO. Our findings are consistent with those of Yabroudi et al. [S6], which indicated that PEMF (5 mT, 50 Hz) combined with progressive resistance exercise offered no additional benefits compared to progressive resistance exercise alone in reducing pain and improving physical functions (KOOS, walking speed and five‐times chair stand test) up to 6‐month post‐intervention in patients with knee OA. Similarly, our results align with those of Dundar et al. [S7], who found that PEMF (0.1 mT, 50 Hz) combined with physical therapy (including hot pack, ultrasound, transcutaneous nerve stimulation and isometric knee exercise) provided no additional benefits for pain reduction, WOMAC score, knee effusion, or ultrasound grading of femoral cartilage degeneration compared to combined physical therapy and sham PEMF therapy in knee OA patients [S7].

However, our results are inconsistent with most, though not all [S8], previous preclinical research. For instance, PEMF has been reported to reduce extracellular matrix (ECM) degradation and enhance chondrogenesis in bone marrow stromal cells [4]. It has also been shown to decrease inflammation, glycolytic activity and catabolism in inflammatory mouse chondrocytes and to reduce cartilage degeneration and osteophyte formation in knee OA mouse models [5, 6]. Additionally, PEMF therapy has been demonstrated to reverse cartilage ECM loss, prevent chondrocyte death and decrease the expression of pro‐inflammatory cytokines and matrix‐degradation enzymes, as well as reduce the decrease in cartilage thickness and synovial inflammation in a temporomandibular joint osteoarthritis rat model [11]. In an osteoporotic osteoarthritis animal model, PEMF therapy effectively prevented cartilage and subchondral bone destruction by upregulating PPARγ and inhibiting chondrocyte apoptosis, inflammation and autophagy [12]. A study in a knee OA Guinea pig model reported that stimulation with PEMF at 75 Hz for 6 h per day over 3 months more effectively reduced cartilage thinning compared to the SHAM or 37 Hz PEMF treatment [13]. These discrepancies may be attributed to differences in PEMF therapy parameters and regimens, as most animal studies have utilized PEMF therapy of varying parameters for hours daily over multiple weeks [5, 6, 11, 12, 13].

A systematic review of RCTs indicated that PEMF therapy reduced pain and improved the quality of life for patients with OA in various joints, although the comparison group was not clearly described [14]. An earlier meta‐analysis involving 16 randomized, placebo‐controlled trials found that PEMF therapy improved pain (pooled WOMAC pain score and VAS pain score), WOMAC stiffness and function scores, but not quality of life in OA patients with short‐term follow‐up [15]. Additionally, PEMF therapy (intensity unspecified, frequency unspecified) was found to be more effective than standard care in reducing joint and soft tissue pain after 14 days in a crossover RCT; however, this study lacked a placebo control and used a PEMF device that heated the skin to temperatures of 40°C–45°C [S9]. Another study demonstrated that PEMF (0.01 mT, 6.8 MHz) reduced pain in patients with early knee OA after daily treatments for 42 days [S10]. Pain levels were assessed daily during the intervention in both studies. PEMF therapy (10 mT, 50 Hz) combined with exercise showed greater improvements in WOMAC pain and stiffness scores, but not VAS pain score, WOMAC physical function and functional test, compared to exercise alone in knee OA patients [36]. Similarly, PEMF therapy (10 mT, 30 Hz) combined with daily exercise significantly improved WOMAC pain, stiffness and total scores but had no significant effects on the WOMAC physical activity score or functional tests compared to exercise alone in early knee OA patients [37]. Six sessions of PEMF therapy (10 mT, 30 Hz) over 3 weeks improved pain score, WOMAC scores and physical function test post‐intervention compared to low‐level laser therapy in early knee OA patients [S11]. The discrepancies compared to our findings may be attributed to differences in study designs, assessment time points, treatment regimens and the intensity and frequency of PEMF signals. The assessment time points in previous studies were generally shorter than in our study, which may have led us to miss the short‐term effects of PEMF therapy. Furthermore, different PEMF exposure parameters may be necessary to effectively stimulate chondrogenesis compared to myogenesis. Future studies should focus on identifying the optimal PEMF parameters to reduce cartilage loss in OA patients.

### 4.10 Strengths, Limitations and Future Studies

The strength of this study lies in its investigation of the muscle‐promoting and OA disease‐modifying effects of PEMF, specifically regarding cartilage thinning and joint space narrowing, in a double‐blind, placebo‐controlled trial with a follow‐up duration of 12‐month post‐intervention. Although our double‐blinded, placebo‐controlled, randomized trial suggests the potential of PEMF therapy to address extensor weakness in this high‐risk population, it has some limitations. First, we estimated the sample size based on pilot data from the first 10 patients. Despite this informed sample size, our study may still lack sufficient power to detect differences, as the variability of outcomes was greater than we initially anticipated. The large but statistically insignificant difference in the percentage change of muscle strength at 12 months post‐PEMF therapy suggests this possibility. Additionally, the single‐centre design of our study may not fully reflect the variability present in the larger knee OA population and may elevate the risk of selection bias. Besides, we focused on patients with early‐to‐moderate knee OA, leaving the effects of PEMF therapy on muscle strength and cartilage degeneration in patients with severe OA unclear. Several areas warrant further exploration. Whereas existing PEMF treatment protocols have demonstrated considerable variability as revealed by a systematic review of systematic reviews (an umbrella review) [S12], the optimal PEMF therapy regimen for clinical use remains undefined, highlighting the need for dose–response investigations tailored to the OA population. Given that many patients with knee OA are elderly, it is essential to understand how comorbidities may influence treatment efficacy. Furthermore, multicentre trials are necessary to establish robust evidence supporting the clinical effectiveness of this therapy.

## Conclusion

In summary, we demonstrated that 8 weeks of PEMF therapy at 30 min per session and three times per week significantly improved knee extensor strength in refractory patients with mild‐to‐moderate knee OA at 6‐month post‐intervention. This suggests its potential to address extensor weakness in this high‐risk population. However, the therapy did not show significant effects on knee flexor strength, lean muscle mass, cartilage thickness, mJSW, lower limb physical functions or knee‐specific PRO up to 12‐month post‐intervention. Further research is needed to identify treatment regimens that may promote structural and functional outcomes in OA patients.

## Funding

This work was supported by the InnoHK initiative of the Innovation and Technology Commission of the Hong Kong Special Administrative Region Government (reference number: ITC RC/IHK/4/7).

## Ethics Statement

The study received approval from the Joint Chinese University of Hong Kong and New Territories East Cluster Clinical Research Ethics Committee (2022.242).

## Conflicts of Interest

The authors declare no conflicts of interest.

## Supporting information


**Figure S1:** Percentage change in knee extension peak torque of the treated limb from baseline at different follow‐up time points. Median and interquartile range are presented.
**Figure S2:**. Percentage change in knee flexor peak torque of the treated limb from baseline at different follow‐up time points. Median and interquartile range are presented.
**Figure S3:**. Changes in lower limb muscle mass of the treated limb with time in the PEMF and SHAM groups (*n* = 30/group). Analysed using a two‐way repeated measures analysis of variance with two treatment groups and four time points. Mean ± 95% confidence intervals.
**Figure S4:**. Changes in cartilage thickness at the medial condyle, intercondylar area and lateral condyle of the femur of the treated limb with time in the PEMF and SHAM groups (*n* = 30/group). Analysed using a two‐way repeated measures analysis of variance with two treatment groups and four time points. Mean ± 95% confidence intervals.
**Figure S5:**. Changes in the minimum joint space width of the treated limb with time in the PEMF and SHAM groups (−*n* = 30/group). Analysed using a two‐way repeated measures analysis of variance with two treatment groups and four time points. Mean ± 95% confidence intervals.
**Figure S6:**. Changes in WOMAC total score with time in the PEMF and SHAM groups (*n* = 30/group). Analysed using a two‐way repeated measures analysis of variance with two treatment groups and four time points. Mean ± 95% confidence intervals.
**Figure S7:**. Changes in 6‐m walk time with time in the PEMF and SHAM groups (*n* = 30/group). Analysed using a two‐way repeated measures analysis of variance with two treatment groups and four time points. Mean ± 95% confidence intervals.
**Figure S8:**. Changes in the number of chair standing repetitions with time in the PEMF and SHAM groups (*n* = 30/group). Analysed using a two‐way repeated measures analysis of variance with two treatment groups and four time points. Mean ± 95% confidence intervals.

**Data S1:** Supporting Information.

## Data Availability

Data are available upon request.
