# Supplementary material for: Pulsed Electromagnetic Field Therapy for Mild‐to‐Moderate Knee Osteoarthritis: A Double‐Blind, Randomized, Placebo‐Controlled Clinical Trial
Source: J Cachexia Sarcopenia Muscle. 2026 Jan 26;17(1):e70199. doi: 10.1002/jcsm.70199 (PMC12834700; doi:10.1002/jcsm.70199)

**Supplementary figure 1**. Percentage change in knee extension peak torque of the treated limb from baseline at different follow-up time points. Median and interquartile range are presented.


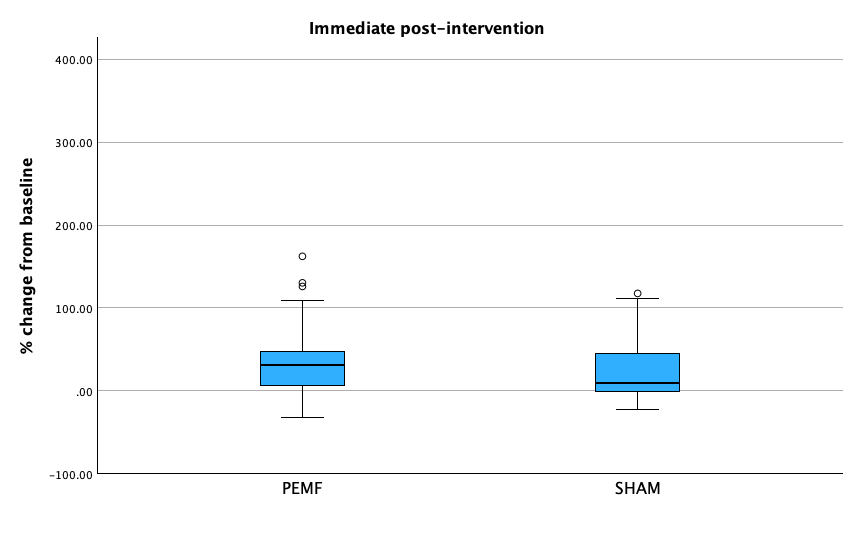


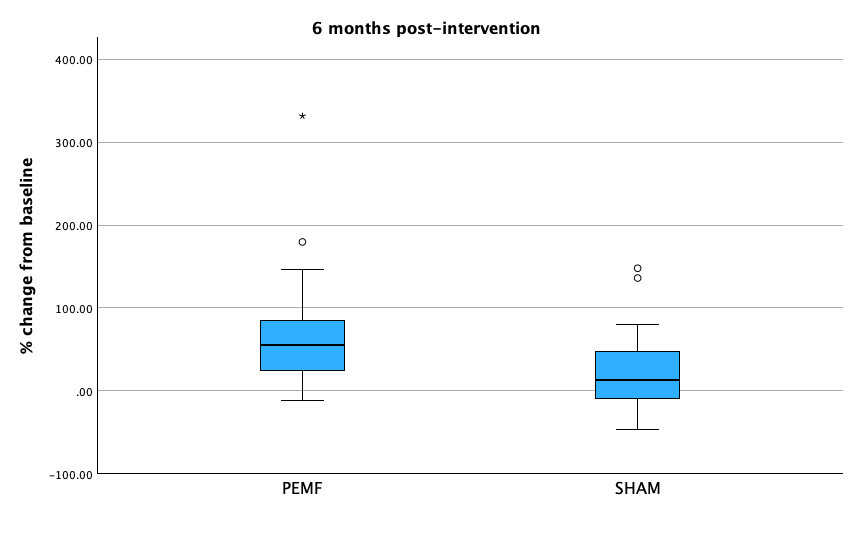


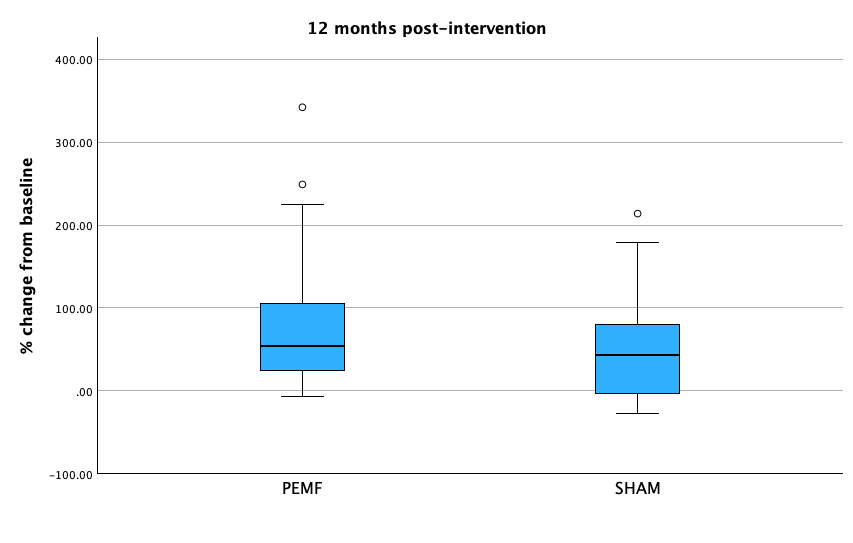


**Supplementary figure 2**. Percentage change in knee flexor peak torque of the treated limb from baseline at different follow-up time points. Median and interquartile range are presented.


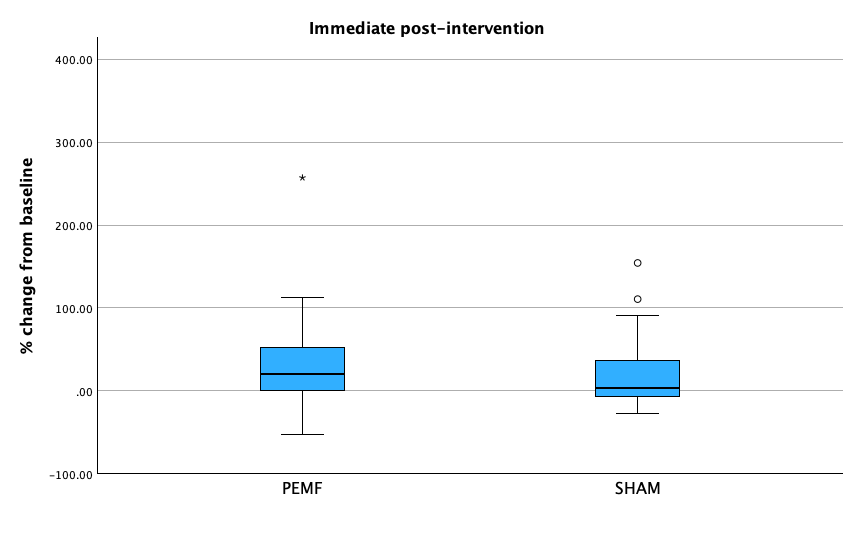


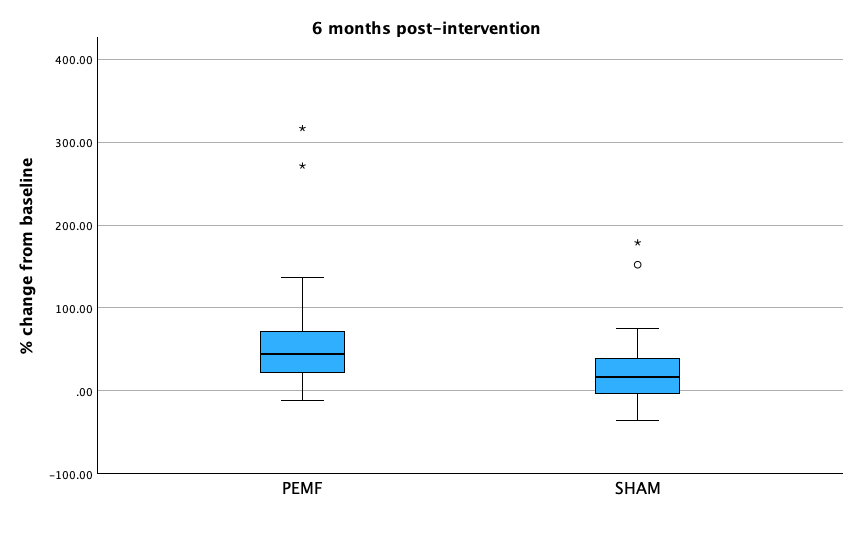


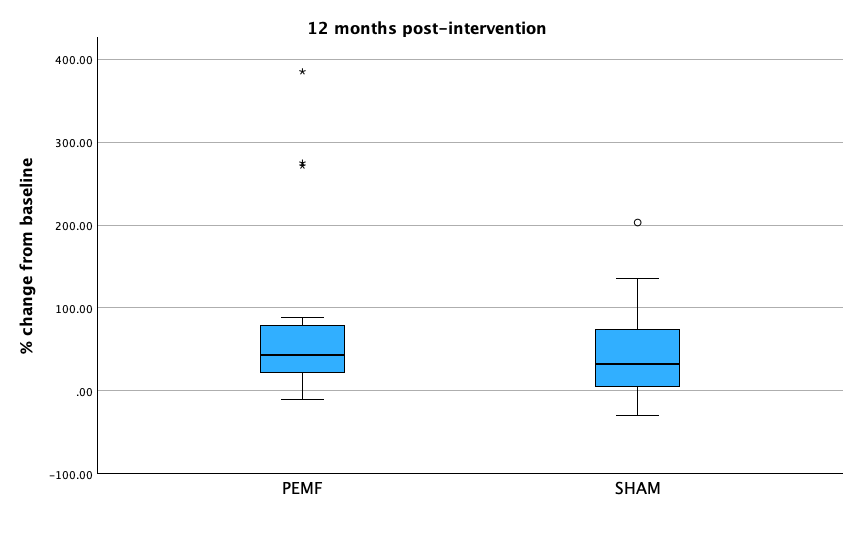


**Supplementary figure 3**. Changes in lower limb muscle mass of the treated limb with time in the PEMF and SHAM groups (n=30/group). Analyzed using a two-way repeated measures analysis of variance with 2 treatment groups and 4 time points. Mean ± 95% confidence intervals.


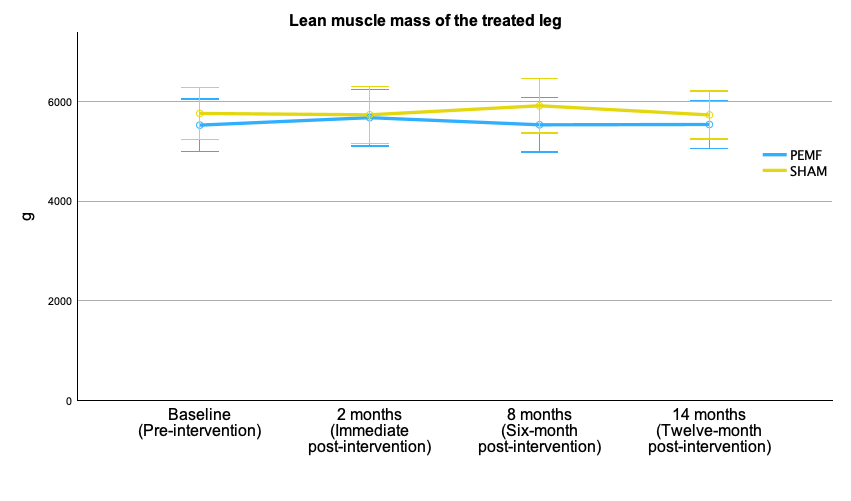


**Supplementary figure 4**. Changes in cartilage thickness at the medial condyle, intercondylar area, and lateral condyle of the femur of the treated limb with time in the PEMF and SHAM groups (n=30/group). Analyzed using a two-way repeated measures analysis of variance with 2 treatment groups and 4 time points. Mean ± 95% confidence intervals.


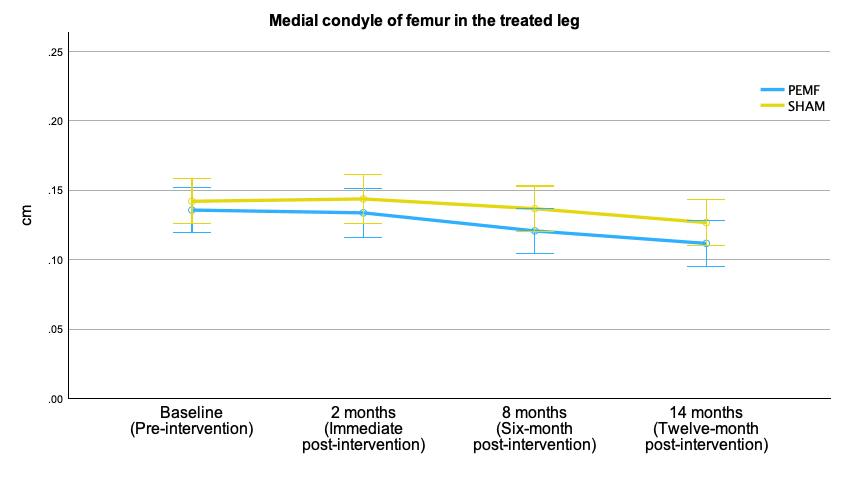


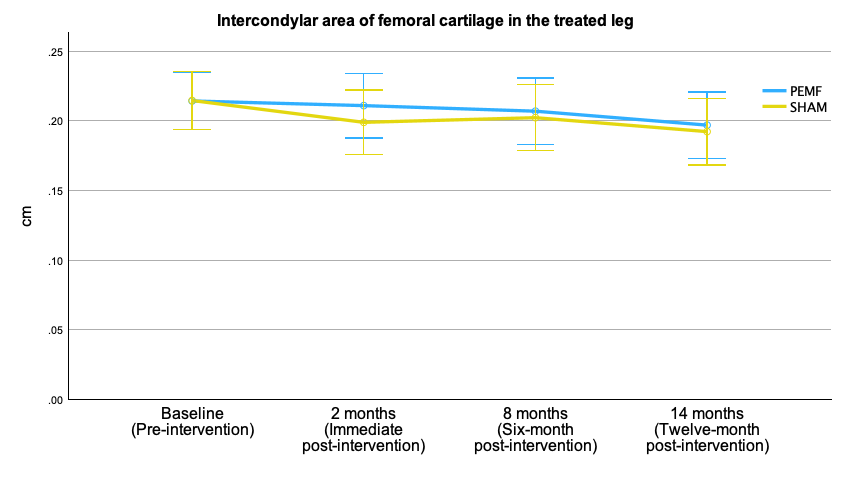


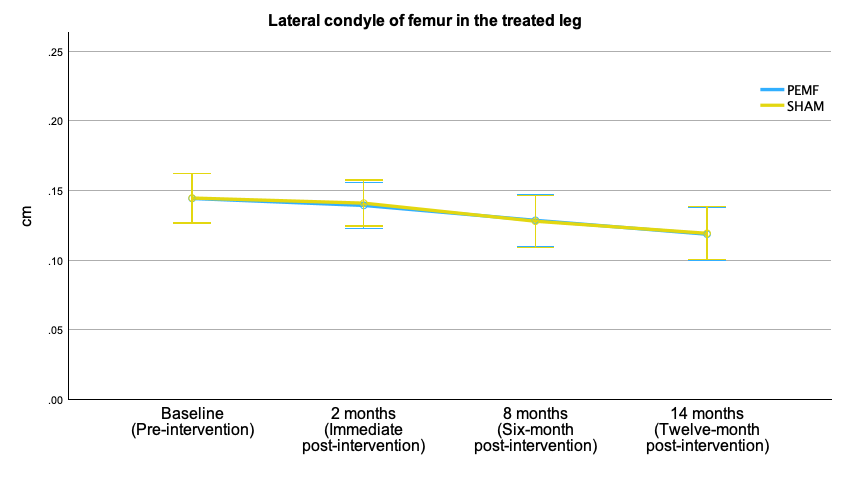


**Supplementary figure 5**. Changes in the minimum joint space width of the treated limb with time in the PEMF and SHAM groups (n=30/group). Analyzed using a two-way repeated measures analysis of variance with 2 treatment groups and 4 time points. Mean ± 95% confidence intervals.


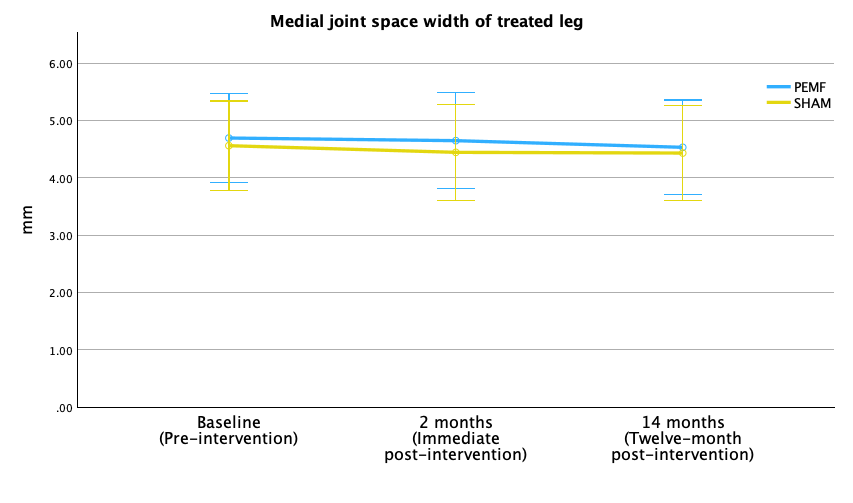


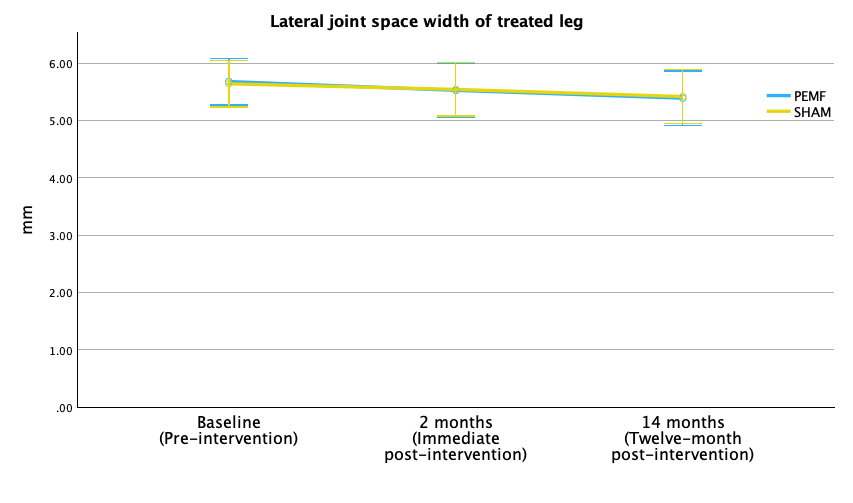


**Supplementary figure 6**. Changes in WOMAC total score with time in the PEMF and SHAM groups (n=30/group). Analyzed using a two-way repeated measures analysis of variance with 2 treatment groups and 4 time points. Mean ± 95% confidence intervals.


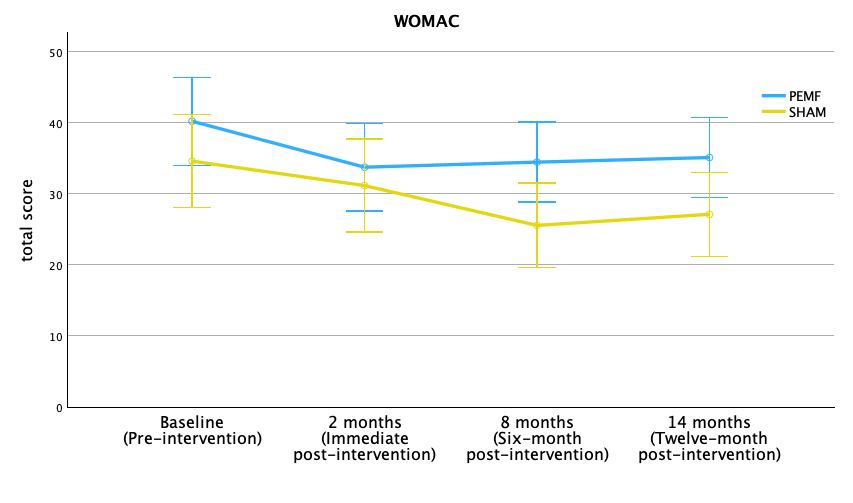


**Supplementary figure 7**. Changes in 6-meter walk time with time in the PEMF and SHAM groups (n=30/group). Analyzed using a two-way repeated measures analysis of variance with 2 treatment groups and 4 time points. Mean ± 95% confidence intervals.


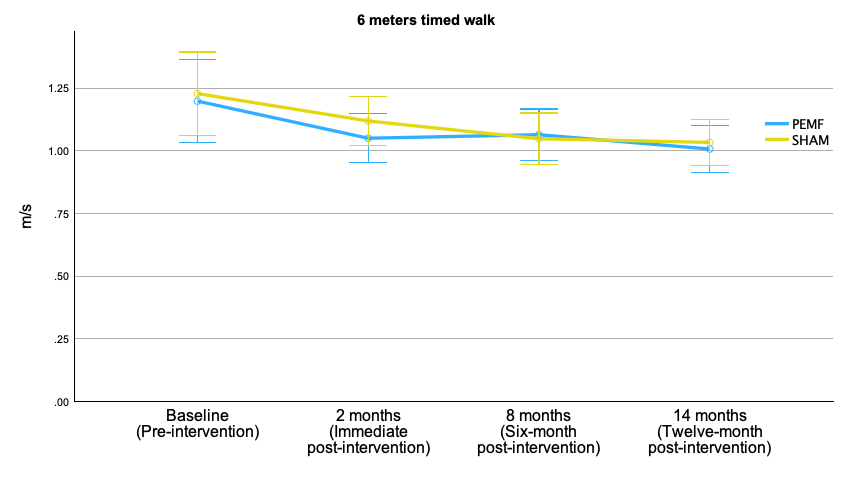


**Supplementary figure 8**. Changes in the number of chair standing repetitions with time in the PEMF and SHAM groups (n=30/group). Analyzed using a two-way repeated measures analysis of variance with 2 treatment groups and 4 time points. Mean ± 95% confidence intervals.


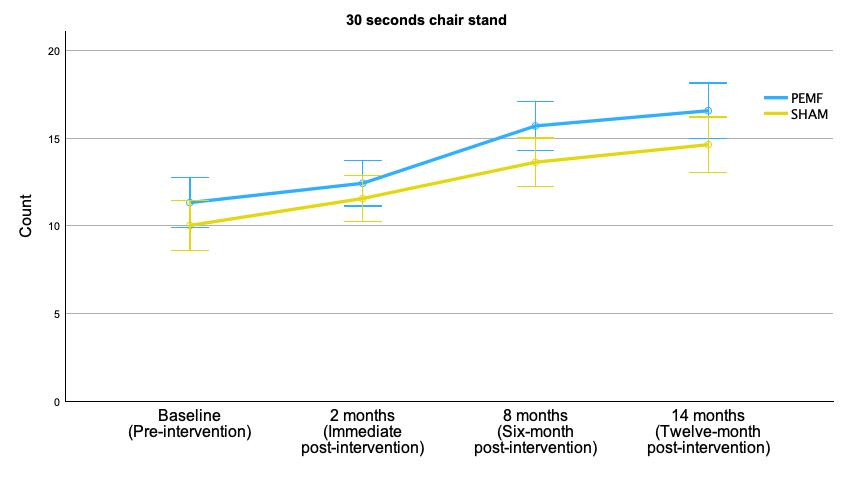

Supplement: Supplementary file 1 — Figure S1: Percentage change in knee extension peak torque of the treated limb from baseline at different follow‐up time points. Median and interquartile range are presented. Figure S2:. Percentage change in knee flexor peak torque of the treated limb from baseline at different follow‐up time points. Median and interquartile range are presented. Figure S3:. Changes in lower limb muscle mass of the treated limb with time in the PEMF and SHAM groups (n = 30/group). Analysed using a two‐way repeated measures analysis of variance with two treatment groups and four time points. Mean ± 95% confidence intervals. Figure S4:. Changes in cartilage thickness at the medial condyle, intercondylar area and lateral condyle of the femur of the treated limb with time in the PEMF and SHAM groups (n = 30/group). Analysed using a two‐way repeated measures analysis of variance with two treatment groups and four time points. Mean ± 95% confidence intervals. Figure S5:. Changes in the minimum joint space width of the treated limb with time in the PEMF and SHAM groups (−n = 30/group). Analysed using a two‐way repeated measures analysis of variance with two treatment groups and four time points. Mean ± 95% confidence intervals. Figure S6:. Changes in WOMAC total score with time in the PEMF and SHAM groups (n = 30/group). Analysed using a two‐way repeated measures analysis of variance with two treatment groups and four time points. Mean ± 95% confidence intervals. Figure S7:. Changes in 6‐m walk time with time in the PEMF and SHAM groups (n = 30/group). Analysed using a two‐way repeated measures analysis of variance with two treatment groups and four time points. Mean ± 95% confidence intervals. Figure S8:. Changes in the number of chair standing repetitions with time in the PEMF and SHAM groups (n = 30/group). Analysed using a two‐way repeated measures analysis of variance with two treatment groups and four time points. Mean ± 95% confidence intervals. [file JCSM-17-e70199-s001.docx]
