# Supplementary material for: Pulsed Electromagnetic Field Therapy for Mild‐to‐Moderate Knee Osteoarthritis: A Double‐Blind, Randomized, Placebo‐Controlled Clinical Trial
Source: J Cachexia Sarcopenia Muscle. 2026 Jan 26;17(1):e70199. doi: 10.1002/jcsm.70199 (PMC12834700; doi:10.1002/jcsm.70199)
Supplement: Supplementary file 2 — Data S1: Supporting Information. [file JCSM-17-e70199-s002.docx]

**Supplementary references**

S1. Wong CJK, Tai YK, Yap JLY, et al. Brief exposure to directionally-specific pulsed electromagnetic fields stimulates extracellular vesicle release and is antagonized by streptomycin: A potential regenerative medicine and food industry paradigm. Biomaterials 2022; 287: 121658.

S2. Huang S, Jin Y, Zhang L, Zhou Y, Chen N, Wang W. PPAR gamma and PGC-1alpha activators protect against diabetic nephropathy by suppressing the inflammation and NF-kappaB activation. Nephrology (Carlton) 2024; 29(12): 858-72.

S3. Kaadan A, Salati S, Cadossi R, Aaron R. Regulation of Inflammatory Responses by Pulsed Electromagnetic Fields. Bioengineering (Basel) 2025; 12(5).

S4. Mohamady HM, Taha MM, Aneis YM, Aldhahi MI, Attalla AF. Effect of Combined Electromagnetic Field and Plantar Flexion Resistance Exercise on Wound Healing in Patients with Venous Leg Ulcers: A Randomized Controlled Trial. Medicina (Kaunas) 2023; 59(6).

S5. Stiller MJ, Pak GH, Shupack JL, Thaler S, Kenny C, Jondreau L. A portable pulsed electromagnetic field (PEMF) device to enhance healing of recalcitrant venous ulcers: a double-blind, placebo-controlled clinical trial. Br J Dermatol 1992; 127(2): 147-54.

S6. Yabroudi MA, Aldardour A, Nawasreh ZH, Obaidat SM, Altubasi IM, Bashaireh K. Effects of the combination of pulsed electromagnetic field with progressive resistance exercise on knee osteoarthritis: A randomized controlled trial. J Back Musculoskelet Rehabil 2024; 37(1): 55-65.

S7. Dündar Ü, Aşık G, Ulaşlı AM, et al. Assessment of pulsed electromagnetic field therapy with Serum YKL-40 and ultrasonography in patients with knee osteoarthritis. Int J Rheum Dis 2016; 19(3): 287-93.

S8. Tan L, Ren Y, van Kooten TG, Grijpma DW, Kuijer R. Low-intensity pulsed ultrasound (LIPUS) and pulsed electromagnetic field (PEMF) treatments affect degeneration of cultured articular cartilage explants. Int Orthop 2015; 39(3): 549-57.

S9. Hackel JG, Paci JM, Gupta S, Maravelas DA, North TJ, Paunescu A. Evaluating Noninvasive Pulsed Electromagnetic Field Therapy for Joint and Soft Tissue Pain Management: A Prospective, Multi-center, Randomized Clinical Trial. Pain Ther 2025; 14(2): 723-35.

S10. Nelson FR, Zvirbulis R, Pilla AA. Non-invasive electromagnetic field therapy produces rapid and substantial pain reduction in early knee osteoarthritis: a randomized double-blind pilot study. Rheumatol Int 2013; 33(8): 2169-73.

S11. Elboim-Gabyzon M, Nahhas F. Laser therapy versus pulsed electromagnetic field therapy as treatment modalities for early knee osteoarthritis: a randomized controlled trial. BMC Geriatr 2023; 23(1): 144.

S12. Markovic L, Wagner B, Crevenna R. Effects of pulsed electromagnetic field therapy on outcomes associated with osteoarthritis : A systematic review of systematic reviews. Wien Klin Wochenschr 2022; 134(11-12): 425-33.
